# Quality of life in adolescents with chronic kidney disease who initiate haemodialysis treatment

**DOI:** 10.1186/s12882-019-1365-3

**Published:** 2019-05-14

**Authors:** Stéphanie Clavé, Michel Tsimaratos, Mohamed Boucekine, Bruno Ranchin, Rémi Salomon, Olivier Dunand, Arnaud Garnier, Annie Lahoche, Marc Fila, Gwenaelle Roussey, Francoise Broux, Jérome Harambat, Sylvie Cloarec, Soraya Menouer, Georges Deschenes, Isabelle Vrillon, Pascal Auquier, Julie Berbis

**Affiliations:** 1 Department of Multidisciplinary Pediatrics, Hôpital de la Timone Enfant, Assistance Publique des Hôpitaux de Marseille, Marseille, France; 2 Aix-Marseille Univ, School of medicine - La Timone Medical Campus, EA 3279: - CEReSS Health Service Research and Quality of Life Center, Marseille, France; 3 Department of Pediatric Nephrology, Hôpital Femme Mere Enfant, Hospices Civils de Lyon, Lyon, France; 4 Department of Pediatric Nephrology, Assistance Publique des Hôpitaux de Paris, University Hospital Necker-Enfants Malades, Paris, France; 5 Department of Pediatrics, Hôpital Félix Guyon, University Hospital La Réunion, Saint-Denis, La Réunion France; 6 Department of Pediatric Nephrology, Children Hospital Toulouse, Toulouse, France; 7 Department of Pediatric Nephrology, Jeanne de Flandre Hospital, University Hospital of Lille, Lille, France; 8 Department of Pediatric Nephrology, hôpital Arnaud-de-Villeneuve, University Hospital of Montpellier, Montpellier, France; 9 Department of Pediatrics, University Hospital of Nantes, Nantes, France; 10 Department of Pediatrics, Pediatric Nephrology and Hemodialysis Unit, University Hospital Charles Nicolle, Rouen, France; 11 Department of Pediatrics, Hôpital Pellegrin-Enfants, University Hospital of Bordeaux, Bordeaux, France; 12 Department of Pediatric Nephrology and Hemodialysis, Clocheville Hospital, University Hospital of Tours, Tours, France; 13 Department of Pediatrics 1, University Hospital of Strasbourg, Hôpital de Hautepierre, Strasbourg, France; 14 Department of Pediatric Nephrology, Assistance publique des Hôpitaux de Paris, University Hospital Robert Debré, Paris, France; 15 Department of Pediatric Nephrology, Hôpital d’Enfants Brabois, Nancy, France

**Keywords:** End-stage renal disease, Adolescents, Initiating haemodialysis, Quality of life, Coping strategies, Bayesian models

## Abstract

**Background:**

To describe the quality of life of adolescents initiating haemodialysis, to determine the factors associated with quality of life, and to assess coping strategies and their impact on quality of life.

**Methods:**

All adolescents initiating haemodialysis between September 2013 and July 2015 in French paediatric haemodialysis centres were included. Quality of life data were collected using the “Vécu et Santé Perçue de l’Adolescent et l’Enfant” questionnaire, and coping data were collected using the Kidcope questionnaire. Adolescent’s quality of life was compared with age- and sex-matched French control.

**Results:**

Thirty-two adolescents were included. Their mean age was 13.9 ± 2.0 years. The quality of life score was lowest in leisure activities and highest in relationships with medical staff. Compared with the French control, index, energy-vitality, relationships with friends, leisure activities and physical well-being scores were significantly lower in haemodialysis population. In multivariate analyses, active coping was positively associated with quality of life and especially with energy-vitality, relationships with parents and teachers, and school performance. In contrast, avoidant and negative coping were negatively associated with energy-vitality, psychological well-being and body image for avoidant coping, and body image and relationships with medical staff for negative coping.

**Conclusions:**

The quality of life of haemodialysis adolescents, and mainly the dimensions of leisure activities, physical well-being, relationships with friends and energy-vitality, were significantly altered compared to that of the French population. The impact of coping strategies on quality of life seems to be important. Given the importance of quality of life and coping strategies in adolescents with chronic disease, health care professionals should integrate these aspects into care management.

## Background

ESRD in children is rare but not exceptional. In 2008, the international incidence of children and adolescents under 20 years old with ESRD was around 9 per million of the age-related population (pmarp), and the most recent incidence in France was 8.7 pmarp in 2015 [1, 2]. In every registry, a higher incidence was found in adolescents. The average European paediatric ESRD incidence was 5.5 cases pmarp in children aged 0–14 years and 8.3 pmarp in children aged 0–19 years [3].

ESRD is a very severe disorder associated with excessive mortality and cardiovascular morbidity, and particular complications occur in children such as impaired growth, and psychosocial adjustment [4]. The aim of any treatment is to minimize renal damage but as kidney function is gradually lost renal replacement therapy (RRT) may be required in the form of haemodialysis, peritoneal dialysis (PD), or renal transplantation [5]. Medical and surgical advances have led to dramatic changes in physical outcomes and substantial improvement in survival rates for children with CKD [6]. However, RRT, especially dialysis, is consuming and invasive, placing high burdens of daily management on children that restrict their physical and social activities [7]. ESRD children must face multiples challenges, including frequent hospitalizations, painful medical procedures, irregular school attendance and restriction of activities. This can have emotional and psychosocial impacts on the children, prompting an interest in thinking about quality of life (QoL), which is increasingly recognized as a key outcome in both clinical and research settings in the paediatric ESRD population. Moreover, adolescence is an important period of transition leading to empowerment, socialization, and the development of personality. Simply surviving is not sufficient, and quality of survival has emerged as a fundamental focus of comprehensive healthcare [8].

Assessments of QoL in CKD children, and particularly in adolescents, are needed to address the psychosocial well-being and to understand the impact of the disease and its treatment. Some studies have evaluated the QoL of children with CKD and ESRD, but populations are heterogeneous, and all treatment modalities are often combined [9–11]. The impact of disease and the management of patients are very different depending on the treatment. Currently in the literature, there is a notable lack of studies focusing on children and adolescents starting haemodialysis. It is therefore useful to examine adolescents with haemodialysis separately for specific care.

Adolescents could use multiple and different ways to cope with CKD and haemodialysis. Coping refers to a mechanism used to regulate the effect of different life stressors, such as ill health, on physiological responses [12]. It is assumed that different coping styles may affect adaptation to illness. Little is known about the influence of coping strategies on QoL in haemodialysis adolescents. Because coping style plays an important role in the psychosocial functioning and QoL of children with chronic conditions, coping has been included in treatment plans and in targeted interventions [13, 14]. Therefore, it is interesting to better identify haemodialysis adolescents who need help with coping strategies.

The aims of this paper are: (1) to describe the QoL of adolescents initiating haemodialysis treatment compared to French age- and sex-matched population; (2) to investigate factors affecting their QoL; and (3) to assess coping strategies and their impact on QoL.

## Methods

### Design

This prospective national study was conducted from September 2013 to July 2015, including the 17 French paediatric haemodialysis centres opened in France during that period. CKD adolescents initiating haemodialysis (regardless of medical history) and their parents were included. The inclusion criteria were as follows: (1) age 11–17 years at the time of evaluation, (2) first haemodialysis for 4 to 6 weeks, (3) capacity to respond to the questionnaire in French, and (4) explicit agreement to participate in the study and signed informed consent of parents or legal representatives.

This study was approved by an ethical research committee (Committee for Personal Protection).

### Data collection

Medical data recorded included: primary diagnosis of kidney disease, age at primary diagnosis and at CKD, medical RRT history, vascular access (arteriovenous fistula/central catheter), number of weekly haemodialysis sessions, duration of a haemodialysis session, number of comorbidities, numbers of treatments, height measurement and laboratory tests (haemoglobin level). Anaemia was defined according to the KDOQI as a haemoglobin level less than the fifth percentile of the reference range after adjusting for age and sex [15].

Social and demographic data were also collected: age and gender, urban or rural residence, time between home and haemodialysis centre, siblings, adolescent’s schooling (yes/not), parent’s age, family structure (separated parents or not), parent’s work situation (at least one of them is working), and financial difficulties (yes or not).

### Measures

#### QoL evaluation

The adolescent’s QoL was assessed using the self-reported questionnaire “Vécu et Santé Perçue de l’Adolescent” (VSP-A), version for 11- to 17-year-olds [16, 17]. It describes QoL in 10 dimensions and an index (mean score): energy-vitality, psychological well-being, relationships with friends, leisure activities, relationships with parents, physical well-being, relationships with teachers, school performance, body image and relationships with medical staff. Each item is answered on a 5-point Likert scale. All scores range between 0 and 100, higher scores indicating better QoL.

Reference values are available for the French general population [18]. For this population, an index bis of VSP-A was calculated, that did not include the dimension of relationships with medical staff

#### Coping strategies evaluation

The cognitive, emotional and social coping of adolescents was assessed using the self-reported questionnaire Kidcope [19]. It is composed of 10 items: distraction, social withdrawal, wishful thinking, self-criticism, blaming others, problem solving, emotional regulation (positive and negative), cognitive restructuring, social support and resignation. The frequency of each coping strategy is answered on a 4-point Likert scale. All scores range between 0 and 3, higher scores indicating a frequent use of coping. The data were dichotomized into absent or present, 0 was scored as absent and 1–3 as present.

These strategies can be grouped into “active coping strategies” (i.e., problem solving, positive and negative emotional regulation, social support, cognitive restructuring), “avoidant coping strategies” (i.e., distraction, social withdrawal, wishful thinking, resignation), and “negative coping strategies” (i.e., self-criticism, blaming others) [20, 21]. Each strategy was considered present if at least one of the items was used. For the score, active coping varied between 0 and 15, avoidant coping between 0 and 12, and negative coping between 0 and 6. Scores were adjusted to a scale of 0 to 10 for each of 3 coping strategies.

### Statistical methods

Descriptive data were expressed as mean and standard deviation (SD) or median and interquartile range (IQR) for quantitative variables, and as counts and percentages.

for qualitative variables.The VSP-A scores were compared with age- and sex-matched controls from the French population.

Mean imputation was used for missing values of VSP-A dimensions if at least half of dimension was informed. This imputation was accounted for in calculating the VSP-A index.

Linear regression analysis was performed to evaluate the relationship between the VSP-A index and different variables. Based on prior knowledge and literature, sex, age at the evaluation, age of CKD, growth failure, haemoglobin and active, avoidant and negative coping strategies were included in the model.

Bayesian statistical models were chosen for all analyses. According to our design, this approach is more robust with small samples and relies on fewer and less strict assumptions of the data [22]. Bayesian estimation makes use of Markov chain Monte Carlo algorithms to iteratively draw random samples from the posterior distribution of the model parameters. Chain convergence was assessed by the Geweke’s Diagnostic Method and the efficiency of the chain [23]. Statistical significance was defined as the 95% credible interval of the posterior probability distribution parameter that did not include the value 0.

The statistical analyses were performed using SPSS software package version 20.0 (SPSS Inc., Chicago, IL, USA) and SAS software, Version 9.4 (SAS Institute, Inc., Cary, NC, 2008).

## Results

### Characteristics of the subjects

One patient declined to participate and 3 centres did not participate in the study. In total, 32 patients were included, and 62.5% (20/32) were boys. Mean age at inclusion was 13.9 ± 2.0 years. The most common cause of CKD was hereditary nephropathy in 40.6% of adolescents (13/32), followed by congenital anomalies of the kidney and urinary tract (CAKUT) in 28.1% of cases (9/32) and glomerulonephritis in 15.6% of cases (5/32). For 75% of patients (24/32), initiation haemodialysis was the first RRT modality. Five adolescents had previous kidney transplant, and 3 had previous PD before starting haemodialysis. Clinically, 28.1% of adolescents (9/32) exhibited growth failure (height ≤ −2SD for age and sex), including 5 not receiving growth hormones (2 with prior renal transplantation). Biologically, 71.9% of adolescents (23/32) had anaemia, all received an erythropoiesis-stimulating agent. The mean number of treatments per patients was 7.1 ± 1.1, and the mean number of comorbidity was 2.9 ± 1.8.

Regarding psychological characteristics, the coping items resignation, cognitive restructuring and distraction were most often used among 91.3% (21/23), 82.6% (19/23) and 80% (20/25) of the sample, respectively. Blaming others and self-criticism were little-used coping items. Active and avoidant coping strategies were used by almost all participants in the study (24/25), contrary to negative coping strategies (8/22).

Most adolescents (21/25) were in school. Among the 4 adolescents who were not attending school, 2 stopped temporarily and 2 definitively. Half of the adolescent lived in the city, and for 30.4% (7/23) of the children, the time between home and the haemodialysis centre was over 60 min. Regarding parents’ characteristics, the parents’ mean age was 46.5 ± 7.4 years, 76% families (19/25) had at least one parent working, and 52% (13/25) had financial issues.

Table 1 shows clinical, psychological and environmental characteristics.

**Table 1:** Clinical, psychological and environmental characteristics of adolescents

*N* = 32	n (%) Mean ± SD Median [Q1; Q3]
*1-Clinical characteristics*	
Age (years)	13.9 ± 2.0
Sex	
Female	12 (37.5)
Male	20 (62.5)
Age at primary diagnosis (years)^a3^	5.8 [0.8; 10.8]
0–5 years	16 (55.2)
6–10 years	7 (24.1)
11–17 years	6 (20.7)
Age at CKD diagnosis (years)^a1^	8.1 [1.9; 13.1]
0–5 years	11 (35.5)
6–10 years	12 (38.7)
11–17 years	8 (25.8)
Delay between primary diagnosis and current HD	8.6 [2.0; 11.7]
Primary diagnosis	
*Congenital nephropathies*	
CAKUT	9 (28.1)
Hereditary nephropathies	13 (40.6)
*Acquired nephropathies*	
Glomerulonephritis	5 (15.6)
Vascular	3 (9.4)
Unknown	2 (6.3)
Medical history	
Renal transplantation and/or HD and/or PD^b^	8 (25)
No	24 (75)
Vascular access of HD	
Arteriovenous fistula	14 (43.8)
Central catheter	16 (50.0)
Arteriovenous fistula and central catheter	2 (6.2)
Number of weekly HD sessions	3.0 [3.0; 3.0]
Duration of a HD session (hour)	4.0 [3.0; 4.0]
Growth failure	
Yes	9 (28,1)
No	23 (71.9)
Anaemia	
Yes	23 (71,9)
No	9 (28.1)
Number of comorbidities	3 [2.0; 3.7]
Neither	2 (6.3)
1	5 (15.6)
≥ 2	25 (78.1)
Number of treatments	7.1 ± 1.1
*2- Psychological characteristics*	
Kidcope	
Distraction^a7^	20 (80)
Social withdrawal^a8^	8 (33.3)
Cognitive restructuring^a9^	19 (82.6)
Self-criticism^a10^	7 (31.8)
Blaming others^a11^	3 (14.3)
Problem solving^a8^	14 (58.3)
Emotional regulation -^a9^	10 (43.5)
Emotional regulation +^a9^	15 (65.2)
Wishful thinking^a9^	14 (60.9)
Social support ^a9^	18 (78.3)
Resignation^a9^	21 (91.3)
^c^ Active coping strategies^a7^	
Utilization strategy Score	24 (96) 3.3 [2.0; 5.7]
^d^ Avoidant coping strategies^a7^	
Utilization strategy Score	24 (96) 3.3 [2.5; 6.2]
^e^ Negative coping strategies^a10^	
Utilization strategy Score	8 (36.4) 0.0 [0.0; 3.3]
*3-Environmental characteristics*	
Age of parents (years)^a7^	46.5 ± 7.3
Family structure^a7^	
Not separated parents	13 (52)
Separated parents	12 (48)
Sibling^a8^	
Yes	22 (91,7)
No	2 (8.3)
Place of residence^a8^	
Urban	12 (50)
Rural	12 (50)
Time between home and HD (minutes)^a9^	60.0 [15–180]
< 20 min	3 (13)
20 to 60 min	13 (56.5)
> 60 min	7 (30.4)
Parental employment^a7^	
Yes	19 (76)
No	6 (24)
Financial situation^a7^	
Difficulty	13 (52)
Not difficulty	12 (48)
Adolescent’s schooling^a7^	
Yes	21 (84)
No	4 (16)

^a^Number of missing data

^b^5 adolescents had kidney transplantation and 3 had peritoneal dialysis

c = Problem solving + emotional regulation + social support + cognitive restructuring

d = Distraction + social withdrawal + wishful thinking + resignation

e = Self-criticism + blaming others

*SD* standard deviation, *CKD* chronic kidney disease, *CAKUT* congenital anomalies of the kidney and urinary tract, *HD* haemodialysis, *PD* peritoneal dialysis

### QoL of haemodialysis adolescents and French reference population

For the haemodialysis adolescents, the highest score was relationships with medical staff (79.3 ± 2.2) followed by body image (76.3 ± 5.1), and the lowest score was leisure activities (29.9 ± 4.6). In comparison with controls, the haemodialysis adolescent’s VSP-A index score were significantly lower: 59.4 ± 2.1 versus 65.9 ± 1.5. Of the 4 dimensions energy-vitality, relationships with friends, leisure activities and physical well-being, haemodialysis adolescent’s scores were significantly lower than those of matched controls.

The results of these scores are detailed in Table 2.

**Table 2:** Self-report VSP-A data for HD adolescents compared with the French population

	HD adolescents	Norms		
	M ± SD	M ± SD	Diff^a^	IC 95%
Energy-vitality	59.9 ± 3.6	69.7 ± 2.6	−9.8	**[− 16.9; − 1.9]**
Psychological well-being	68.2 ± 4.2	72.9 ± 2.9	−4.7	[− 12.4; 3.6]
RS with friends	56.6 ± 4.8	67.9 ± 3.4	− 11.3	**[− 21.1; − 1.5]**
Leisure activities	29.9 ± 4.6	55.7 ± 3.2	− 25.8	**[− 34.6; − 16.1]**
RS with parents	65.8 ± 4.6	59.4 ± 3.2	6.4	[− 2.3; 15.6]
Physical well-being	50.5 ± 3.7	72.2 ± 2.6	− 21.7	**[− 28.9; − 14.1]**
RS with teachers	66.3 ± 5.8	57.4 ± 4.1	8.9	[−2.4; 20.9]
School performance	60.8 ± 4.1	62.7 ± 2.9	− 1.9	[− 9.1; 5.7]
Body image	76.3 ± 5.1	75.4 ± 3.6	0.9	[− 9.1; 11.7]
Index bis	59.4 ± 2.1	65.9 ± 1.5	−6.5	**[−10.6; −2.2]**
RS with medical staff	79.3 ± 2.2			
Index	61.4 ± 2.1			

*M* mean, *SD* standard deviation, *HD* haemodialysis, *IC 95%* 95% confidence interval of difference

^a^ Difference HD adolescents score - Norms score (sex- and gender-matched)

*RS* relationships

Bold value = significant value

### Factors associated with QoL in haemodialysis adolescents

In the univariate analysis, no significant association was found between clinical, psychological and environmental variables and the VSP-A index.

In the multivariate model with the variables identified a priori, current age, age at CKD diagnosis, and active coping were significantly associated with the VSP-A index. QoL decreased with age, and early CKD diagnosis was associated with worse QoL. Adolescents using active coping had better QoL (Table 3).

**Table 3:** Factors associated with the VSP-A index in haemodialysis adolescents

	Index VSP-A	Univariate model	Multivariate model*
	Mean ± SD	β [IC 95%]	β [IC 95%]
1-Adolescents			
Gender			
Male^a^	61.8 ± 2.5		
Female	60.8 ± 4.0	− 0.9 [−7.7; 7.5]	− 1.4 [−8.3; 5.3]
Age		−0.6 [−2.7; 1.4]	**−3.5 [−5.2; − 1.9]**
Primary diagnosis			
Congenital^a^	59.6 ± 2.4		
Acquired	65.2 ± 4.3	5.6 [−2.8; 14.1]	–
Age at primary diagnosis		0.0 [−0.7; 0.8]	–
11–17 years^a^	63.4 ± 4.7		
6–10 years	62.5 ± 6.6	− 0.9 [−13.8; 12]	
0–5 years	60.6 ± 5.5	−2.8 [− 13.6; 8.1]	
Age at CKD diagnosis		0.4 [−0.3; 1.2]	**0.8 [0.2; 1.4]**
11–17 years^a^	66.9 ± 3.9		
6–10 years	66.1 ± 5.1	−4.8 [−14.8; 5.1]	
0–5 years	57.5 ± 5.1	−9.4 [−19.5; 0.7]	
Delay between primary diagnosis and HD		−0.2 [− 0.9; 0.6]	
Medical history			
No^a^	61.9 ± 2.4		
Renal transplantation and/or HD and/or PD	59.9 ± 4.7	−2.1 [−11.3; 7.1]	–
Growth failure			
No^a^	62.8 ± 2.4		
Yes	58.1 ± 4.5	−4.7 [−13.4; 4.0]	−0.7 [−7.6; 6.2]
Anaemia			
No^a^	63.1 ± 3.8		
Yes	60.7 ± 4.5	−2.3 [−11.2; 6.5]	−1.5 [−8.3; 5.3]
Number of comorbidities		−1 [−3.5; 1.4]	–
Number of treatments		0.4 [− 3.4; 4.0]	–
Active coping		1.3 [−0.3; 2.9]	**2.9 [1.0; 4.7]**
Avoidant coping		−0.0 [−1.6; 1.6]	−1.1 [−2.5; 0.4]
Negative coping		−0.2 [−2.3; 1.9]	− 1.4 [− 3.2; 0.4]
2-Environmental			
Age of parents		−0.4 [− 0.9; 0.1]	–
Family structure			
Separated parents^a^	60.1 ± 2.9		
Not separated parents	62.3 ± 4.1	2.3 [−5.6; 10.3]	–
Siblings			
No^a^	70.2 ± 6.8		
Yes	60.2 ± 7.2	−9.9 [−24.2; 4.0]	–
Place of residence			
Rural^a^	64.3 ± 2.8		
Urban	57.9 ± 4.0	−6.4 [−14.1; 1.6]	–
Time between home and HD		0.1 [−0.0; 0.1]	–
< 20 min^a^	53.1 ± 5.1		
20 to 60 min	60.7 ± 5.7	7.6 [−3.6; 19]	
> 60 min	61.6 ± 6.1	8.5 [− 3.5; 20.6]	
Parental employment			
No^a^	63.9 ± 4.0		
Yes	60.3 ± 4.7	− 3.6 [− 12.8; 5.5]	–
Financial situation			
Difficulty^a^	61.3 ± 2.9		
Not difficulty	61.1 ± 4.1	−0.3 [−8.1; 7.8]	–
Adolescent’s schooling			
No^a^	58.2 ± 4.9		
Yes	54.7 ± 5.4	3.6 [−7.2; 14.3]	–

*β* correlation coefficient, *IC 95%* 95% confidence interval, *SD* standard deviation, *CKD* chronic kidney disease, *HD* haemodialysis, *PD* peritoneal dialysis

^a=^ reference category ^*^Multivariate model is done on 21 patients

Bold values = statistically significant value

Exploring the link between each dimensions of VSP-A and coping strategies, active coping was positively linked with energy-vitality, relationships with parents and teachers, and school performance. Avoidant coping was negatively linked with energy-vitality, psychological well-being, and body image, and negative coping was negatively linked with body image and relationships with medical staff (Table 4).

**Table 4:** Relationship between coping strategies and dimensions of VSP-A

Multivariate model										
*N* = 21	**VSP-A** **Energy** β [IC 95%]	**VSP-A** **Psycho** β [IC 95%]	**VSP-A** **Friends** β [IC 95%]	**VSP-A** **Activities** β [IC 95%]	**VSP-A** **Parents** β [IC 95%]	**VSP-A** **Physical** β [IC 95%]	**VSP-A** **Teachers** β [IC 95%]	**VSP-A** **School** β [IC 95%]	**VSP-A** **Body** β [IC 95%]	**VSP-A** **Medical** β [IC 95%]
Active coping	**4.1** **[0.2; 7.9]**	0.7 [−4.2; 5.5]	4.6 [−0.6; 9.7]	−1.6 [−7.1; 3.9]	**6.8** **[0.8; 12.7]**	−1.1 [−5.7; 3.6]	**8.3** **[2.9; 13.5]**	**4.8** **[1.3; 8.3]**	0.0 [−3.5; 3.5]	2.2 [−1.2; 5.6]
Avoidant coping	**−4.1** **[−7.1; − 1.0]**	**−4.6** **[−8.5; − 0.8]**	2.1 [−2.0; 6.2]	−0.7 [− 5.1; 3.6]	−1.5 [− 6.2; 3.3]	0.1 [−3.6; 3.8]	3.1 [− 1.2; 7.3]	1.3 [− 1.5; 4.1]	**−7.1** **[− 9.9; − 4.3]**	1.1 [−1.6; 3.8]
Negative coping	−1.5 [− 5.2; 2.2]	0.4 [−4.2; 5.1]	−0.0 [− 5.0; 5.0]	2.1 [− 3.2; 7.4]	− 2.8 [− 8.5; 2.9]	0.4 [− 4.1; 4.9]	−4.9 [− 10.1; 0.2]	−0.1 [− 3.5; 3.2]	**−3.9** **[− 7.3; − 0.5]**	**−3.6** **[− 6.8; − 0.3]**

Result adjusted on gender, age, age of chronic kidney disease, growth failure, haemoglobin

*β* correlation coefficient, *IC 95%* 95% confidence interval

VSP-A = “Vécu et Santé Perçue de l’Adolescent”

Energy = energy-vitality; Psycho = psychological well-being; Friends = relationships with friends; Activities = leisure activities; Parents = relationships with parents; Physical = physical well-being; Teachers = relationships with teachers; School = school performance; Body = body image; Medical = relationships with medical staff

Bold value = statistically significant value

## Discussion

The current study can be considered representative of French haemodialysis adolescents, including 17 paediatric nephrology centres in the country. The French national network REIN (Réseau Epidémiologique et Information en Néphrologie), which describes the incidence and the prevalence of RRT, records more patients aged 10–17 years initiating haemodialysis than our study, with 34 in 2014 and 41 in 2015 [2, 24]. But our study includes only paediatric centres, while the REIN report also includes adolescents cared for in adult centres.

While waiting for a transplant, the initial treatment modality varies with age and relies on haemodialysis and PD. In our cohort, 75% of adolescents initiated RRT with haemodialysis. In Europe three-quarters of adolescents aged 15–19 years start with haemodialysis, unlike younger children aged 0–14 years who start with PD [1].

We report that severe issues of haemodialysis in adolescents altered QoL in specifics dimensions compared to the French population. QoL among the adolescents with haemodialysis was lower in almost all dimensions, but these differences were significantly for physical dimensions: energy-vitality, physical well-being, and social dimensions: relationships with friends and leisure activities. Previous studies have reported that QoL scores in physical and social subscales were significantly lower in dialysis children than in healthy controls [7, 25–28]. Moreover, one study reported significantly lower scores in physical dimensions in dialysis children than in healthy children, but no statistically significant differences were reported for the social subscales [11]. On the other hand, school performance and psychological well-being were lower in haemodialysis adolescents than in the French population, but these differences were not significant. It can be attributed to a relatively small sample and thus a lack of statistical power. Previous studies have reported that the school functioning among dialysis children was significantly lower than in healthy children [11, 27, 28]. Transportation and many hours in hospitals make it difficult or even impossible to integrate with school peers. Murray et al. reported that 25% of young adults leave school at 16 years of age [29]. This school absenteeism leads to isolation amongst friends and can affect psychological well-being. Moreover, similar to other adolescents with a chronic health condition, ESRD adolescents face challenges to their psychological well-being, including body image, due to medication effects (steroids or cyclosporine), procedures (dialysis access) or short stature [30].

By contrast, in our work, relationships with parents and teachers were higher in haemodialysis adolescents that in French controls. These differences were close to reaching statistical significance, again likely because of the lack of power in the study. Children with chronic illnesses are heavily dependant on parental support in many areas of their lives. To face the complexity of care, parent adopt caregiver role in addition to their parental role. This relationship is even more important to preserve because the quality of care that they provide is an important determinant of outcomes for their children [31].

Direct comparison with other studies is difficult because most used different assessment measures, which may contribute to the divergence of data, and the populations are heterogeneous. In the literature there are few studies that evaluate QoL in dialysis children. Because of the small effective, QoL was compared in CKD children treated with various methods (haemodialysis/PD/renal transplantation) or at different stages (predialysis vs RRT) [7, 11, 26, 32]. Two studies [25, 28] assessed QoL in patients undergoing dialysis (haemodialysis /PD) and renal transplantation, whereas another [9] assessed QoL in children undergoing dialysis (haemodialysis /PD) and conservative treatment. One paper measured QoL in a homogeneous population: haemodialysis in children aged 2 to 18 years [27]. The results of this study are along the same lines as ours, but the analysis does not address the same age group, and comparisons with healthy children are not sex- and age-matched. Altogether, there is a lack of studies on adolescents undergoing haemodialysis only.

The other important results of our study are the potential determinants of QoL, in particular coping strategies, which were poorly documented in previous studies. We investigated several parameters that were linked to QoL in the multivariate analysis in order to help clinicians identify children with a high risk of early altered QoL.

The short stature was associated with a negative impact on QoL [33]. The patient’s appearance of a short stature or bone deformities, which appear mainly during puberty, is a significant element of self-acceptance [27]. Short stature may foreshadow poor long-term social outcomes that have been observed by a number of research groups [33–35]. Anaemia was also linked to poor outcomes and poor QoL in CKD children [36]. With respect to the literature, our study also reports a negative correlation, although not significant, between QoL and growth failure and anaemia. In contrast, older age and a longer duration of CKD were significantly associated with a worse VSP-A index. Lopes et al. showed a progressively negative effect of the age of children on the patient’s QoL, especially in children aged > 8 years [7]. Marciano et al. presented similar results, with a significant relationship between the age of children older than 10 years old and a lower global QoL score in multivariate analysis [26].

Finally, our study suggested that QoL among haemodialysis adolescents would be significantly linked with coping strategies. The concept of coping allows in response to a stressor, to use cognitive, emotional and behavioural strategies [12]. Different strategies of coping may influence adaptation to illness. Previous research has described coping in other paediatric populations, in particular patients with coeliac disease [20], chronic fatigue syndrome [21], diabetes [37], and asthma [38]. However, to our knowledge, no study has previously investigated these coping strategies in haemodialysis adolescents. In our study, adolescents used multiple ways to cope with haemodialysis treatment. The most frequently used coping strategies included the 2 avoidant coping strategies of resignation and distraction and the 2 active coping strategies of cognitive restructuring and social support. Garralda et al. reported in children with chronic fatigue syndrome that resignation was one of the most frequently applied coping strategies [21]. It has been suggested that the allocation of resignation to the category of avoidant coping strategies should be revised, as resignation might simply reflect an adolescent’s acceptance of a chronic disease [21]. Negative coping strategies (blaming others and self-criticism) were identified as coping strategies in people with emotional disorders [21]. In our study, these coping strategies seem to be less used, which may be related to a less-altered psychological well-being.

In multivariate analysis, active coping was positively associated with the VSP-A index. In contrast, adolescents using avoidant and negative coping seemed to have worse QoL, but these differences were not statistically significant. To explore this relationship between coping strategies and QoL among haemodialysis adolescents, we examined the impact of all coping strategies on each dimension of VSP-A in a separate multivariate analysis. First, we observed that no coping strategy was significantly associated with the following 3 dimensions: relationships with friends, leisure activities and physical well-being. Since these 3 dimensions were the most altered in our study, we can consider that improving the use of coping strategies would improve the altered QoL among haemodialysis adolescents in these domains. On the other hand, active coping significantly improved relationships with parents and teachers, which are the 2 highest QoL dimensions in haemodialysis adolescents, in addition to school performance. Zimmer-Gembeck et al. have reported that adolescents with positive relationships with families and teachers used more active coping behaviours in response to problems at both home and school [39]. However, if their results are consistent with ours, it appears that the link between coping and QoL is complex to explore in a cross-sectional study. A further longitudinal approach may help us to better understand this mechanism. In contrast, negative coping significantly altered body image and relationships with medical staff. This result may suggest a poor self-image of this group, and the medical staff is on the front line. In this view, the medical staff is highly associated with, and even responsible for, the disease. Avoidant coping was negatively associated with mental dimensions of QoL: energy-vitality, psychological well-being and body image. In the literature, avoidant coping is indeed described as a psychological risk-factor for adverse responses to stressful events [40]. Our findings should encourage healthcare professionals to integrate an assessment of patients’ coping style into the patients’ care and to help them to implementing healthy coping strategies through targeted support.

To go further in understanding children adaptation mechanisms with CKD and haemodialysis, future studies could explore the impact of the level of resilience (characterized by good outcomes despite risk or adversity to development [41]), might have in their quality of life and coping strategies. Optimism, social support or self-efficacy could be some resilience resources and mechanisms which would be relevant to modeling in order to explain patients’ quality of life levels.

Our study had some limitations. The small group of patients probably reduced the power of our study. The limited number of patients may be related to less haemodialysis initiations during the 2 years of the study, or to a lack of participation of some paediatric haemodialysis centres. Nevertheless, our study accounts for one of the largest and most homogenous studies published thus far. Only two other studies included at most 25 haemodialysis children, but they were 2 to 18 years old [9, 27], while in our study, the population is homogenous: 32 adolescents with haemodialysis treatment only. Only one study measured QoL in haemodialysis children only [27] . Futhermore, our statistical methods were adapted to small groups.

Another limitation could be an early assessment of quality of life, and represents QoL of the advanced CKD stage rather than the RRT process. But data were collected 4 to 6 weeks after the start of haemodialysis. The transition with haemodialysis immediately affects the patient’s daily life with sessions at the hospital at least 3 times a week for 4 h or more, with painful medical procedure, irregular school attendance and restriction of activities. We believe that the QoL is already being impacted.

Finally, if the comparison between haemodialysis patients and the controls from the French population was age- and sex-matched, the socioeconomic profile was not considering. Unfortunately, the available data did not allow us to compare the repartition of socioeconomic status between the two groups.

## Conclusions

QoL of haemodialysis adolescents, and mainly the dimensions of leisure activities, physical well-being, relationships with friends and energy-vitality, were significantly altered when compared to that of a French population. Older age at the initiation of haemodialysis and early CKD diagnosis seem to decrease QoL. Moreover, active coping seems to improve QoL, unlike avoidant and negative coping. Hence, healthcare professionals should integrate knowledge of coping processes into care management in order to support positive adaptation and QoL.

## Abbreviations

CAKUT: congenital anomalies of the kidney and urinary tract
CI: confidence interval
CKD: chronic kidney disease
ESRD: end-stage renal disease
IQR: interquartile range
KDOQI: Kidney Disease Outcomes Quality Initiative
PD: peritoneal dialysis
Pmarp: per million of the age-related population
QoL: quality of life
REIN: Réseau Epidémiologique et Information en Néphrologie
RRT: renal replacement therapy
SD: standard deviation
VSP-A: Vécu et Santé Perçue de l’Adolescent
